# Editorial: How children learn from parents and parenting others in formal and informal settings: international and cultural perspectives, volume II

**DOI:** 10.3389/fpsyg.2025.1724813

**Published:** 2025-11-04

**Authors:** Claudio Longobardi, Yvette Renee Harris

**Affiliations:** ^1^Department of Psychology, University of Turin, Turin, Italy; ^2^The Department of Psychology, Miami University, Oxford, MS, United States

**Keywords:** parenting practices, cross-cultural perspectives, child development, family dynamics, emotional and social learning

## Introduction

The family unit is recognized as the primary context for children's development and learning. Within the family, children acquire various skills and knowledge, learn to read emotions, manage social relationships, and adopt moral and cultural values. Parenting practices, caregiving arrangements, and everyday family interactions constitute the first “classroom,” the initial setting in which children's developmental trajectories are shaped (Frosch et al., 2021). Recently, researchers have highlighted the importance of considering both formal and informal learning experiences and frameworks for understanding these processes, as well as examining them across cultures and internationally (Weller et al., 2025).

The first volume of this Research Topic (Harris and Longobardi, 2020) emphasized the fundamental importance of parents and caregivers as mediators of children's cognition and emotion, presented contributions from various cultural contexts, and illustrated how different parenting practices ultimately point to one thing: children learn from parents, but they also learn about parenting in ways that are both universal and culturally specific. Volume II builds and extends on this discussion by including 13 additional articles that address new concerns and offer new ideas about how children learn within family and caregiving contexts, and the role of family contexts on social and clinical outcomes.

In this editorial for Volume II we briefly introduce these contributions. These contributions are organized into four thematic clusters: *(1) family structures and caregiving roles; (2) parenting, emotion, and child adjustment*; (*3) cultural and comparative views;* and *(4) family conflict, wellbeing, and healthy lifestyles*. In sum, these contributions illustrate the various ways in which children learn, grow and thrive within diverse family contexts around the world.

## Family structures and caregiving roles

Family forms vary widely across societies, resulting in diverse caregiving roles for children. Several articles in this issue examine how family structures may influence child development.

Leung and Shek studied children's roles in single-mother families from low-income backgrounds and discussed the duality of filial responsibility, noting that it may promote resilience and maturity but can become burdensome when roles exceed the child's developmental capacity. The authors identified maternal warmth as a modifiable factor that can mitigate the risks associated with excessive responsibility.

Feng et al. examined the role of older sisters in Chinese families after the abolition of the one-child policy. These siblings act as “secondary parents,” contributing to the socialization and academic development of younger children. The study highlights the significance of informal caregiving roles, showing how families can reallocate parental resources in culturally specific ways.

Tang et al. investigated multigenerational and “skip-generation” families, where grandparents are primary caregivers. Their findings showed that the influence on children's non-cognitive skills, such as perseverance and self-regulation, underscores the importance of extended family structures for developmental outcomes.

Finally, Ergin and Demirbaş adapted the Maternal Gatekeeping Scale for Turkish culture, using parents of infants. Their validation of both mother and father forms provides researchers with a reliable tool to examine maternal behaviors that facilitate or restrict fathers' engagement in early caregiving. This is an important contribution to understanding how gatekeeping roles during infant caregiving affect overall developmental outcomes.

## Parenting practices, emotions, and child adjustment

A second group of studies highlights how parenting practices shape children's socio-emotional development, often in subtle yet far-reaching ways.

Modirrousta and Harris showed that parents from minority groups actively scaffold theory of mind in children diagnosed with autism spectrum disorder (ASD), using culturally grounded strategies that extend support beyond clinical settings into everyday family life.

WenLi et al., linked parenting styles to maladaptive emotional regulation and social withdrawal, while reaffirming the protective value of authoritative parenting in fostering resilience. Their findings underline what developmental science has long suggested: warmth and responsiveness provide children with the emotional foundation to manage challenges effectively.

Wen et al. demonstrated that supportive literacy practices at home reduce the likelihood of reading difficulties such as dyslexia, emphasizing the preventive power of family engagement in early education.

Beck illustrated how mothers' scaffold moral understanding through ordinary conversations about fairness, justice, and empathy, showing that moral reasoning develops not only in formal lessons but also in playful, everyday exchanges.

Finally, Fabris et al. revealed how parental rejection contributes to adolescents' vulnerability to generalized anxiety disorder, with alexithymia mediating this link. This study shows how the absence of emotional acceptance can undermine children's capacity to recognize and process feelings, creating pathways toward clinical symptoms.

Taken together, these contributions show that parenting practices do more than shape immediate behavior: they lay the groundwork for children's emotional wellbeing, cognitive strengths, and moral growth.

## Cultural and comparative perspectives

One of the defining features of this Research Topic is its international scope.

Veraksa et al. presented a comparative study of executive functions among children in Japan and Russia. Results revealed both cultural differences and similarities, illustrating how sociocultural contexts influence cognitive control and flexibility. This work underlines the necessity of cross-cultural research in developmental psychology, as it prevents the overgeneralization of findings based on single-cultural samples and enriches theoretical models with a diversity of perspectives.

## Family conflict, wellbeing, and healthy lifestyles

A fourth group of studies examines how family conflict, wellbeing, and lifestyle factors intersect in children's development.

Kong et al. analyzed the impact of parental conflict and family functioning on socially aversive emotions in adolescents. Findings showed that high levels of conflict and low levels of cohesion predict negative emotional outcomes, reinforcing the idea that the quality of the family environment plays a critical role in adolescents' adjustment.

Song and Ge investigated the relationship between parental exercise consciousness and physical activity among 9–11-year-old children. They showed that parents' awareness, attitudes, and willingness regarding physical exercise strongly predict children's activity levels, with important implications for promoting healthy lifestyles in childhood.

These contributions remind us that learning within families extends beyond cognitive and emotional skills. Children also acquire habits, routines, and health behaviors from their parents, shaping their long-term wellbeing.

## General reflections and future directions

The 13 contributions collected in this volume present a consistent and compelling message: children's development is shaped by parents and caregivers in ways that are multifaceted, culturally situated, and deeply relational. Learning within the family extends far beyond the acquisition of language, literacy, and cognitive skills; it includes the cultivation of emotional regulation, moral reasoning, social identities, and health-related habits. Parents, siblings, grandparents, and members of the wider community serve not only as models but also as guides and co-constructors of children's developmental pathways.

Methodologically, the articles highlight the value of employing diverse approaches, quantitative, qualitative, and mixed-methods, while emphasizing the ongoing need for longitudinal designs that can trace how parenting practices, caregiving roles, and family conflict exert long-term influences on children's lives. At the same time, the contributions remind us that research on family dynamics cannot be separated from its social and historical context. Findings from China, Turkey, Italy, Japan, Russia, and other settings demonstrate the benefits of comparative and cross-national inquiry, demonstrating how cultural diversity broadens and refines theoretical models.

The practical implications are equally clear. Supporting parents through training, targeted interventions, and policy measures is essential to promoting children's wellbeing. Evidence indicates that programs encouraging parental warmth, reducing conflict, redistributing caregiving responsibilities, and nurturing healthy family routines can have lasting positive effects. Yet the work is not complete. Expanding research to include underrepresented populations and contexts, particularly in the Global South, will be crucial to ensuring that developmental science captures the full spectrum of children's lived experiences and informs practices that are both equitable and effective.
